# Exopolysaccharide-producing *Lacticaseibacillus paracasei* strains isolated from kefir as starter for functional dairy products

**DOI:** 10.3389/fmicb.2023.1110177

**Published:** 2023-02-24

**Authors:** Ana Agustina Bengoa, María Teresa Dueñas, Alicia Prieto, Graciela L. Garrote, Analía G. Abraham

**Affiliations:** ^1^Centro de Investigación y Desarrollo en Criotecnología de Alimentos (CIDCA) (CONICET-UNLP-CIC), Buenos Aires, Argentina; ^2^Dpto. de Química Aplicada, Facultad de Química, Universidad del País Vasco (UPV/EHU), San Sebastián, Spain; ^3^Grupo de Sistemas Microbianos e Ingeniería de Proteínas, Dpto. de Biotecnología Microbiana y de Plantas, Centro de Investigaciones Biológicas Margarita Salas, Consejo Superior de Investigaciones Científicas, Madrid, Spain; ^4^Area Bioquímica y Control de Alimentos (Dto de Ciencias Biológicas - Facultad de Ciencias Exactas, UNLP), Buenos Aires, Argentina

**Keywords:** exopolysaccharides, *Lacticaseibacillus paracasei*, kefir, fermented milks, functionality

## Abstract

Exopolysaccharides (EPS) produced by lactic acid bacteria are molecules of great interest for the dairy food industry. *Lacticaseibacillus paracasei* CIDCA 8339, CIDCA 83123, and CIDCA 83124 are potentially probiotic strains isolated from kefir grains whose EPS-production on MRS broth is dependent on incubation temperature. The aim of the present work is to evaluate the effect of fermentation temperature on the characteristics of EPS produced in milk by *L. paracasei* strains and the consequent impact on the rheological properties of the fermented products. Additionally, the protective effect of these EPS against *Salmonella* infection was evaluated *in vitro*. Acid gels with each strain were obtained by milk fermentation at 20°C, 30°C, and 37°C evidencing for all the strains a reduction in growth and acidification rate at lower temperature. *Lacticaseibacillus paracasei* CIDCA 83123 showed low fermentation rate at all temperatures requiring between 3 and 8 days to obtain acids gels, whereas CIDCA 8339 and 83124 needed between 24 and 48 h even when the temperature was 20°C. Fermentation temperature led to changes in crude EPS characteristics of the three strains, observing an increase in the relative amount of the high molecular weight fraction when the fermentation temperature diminished. Additionally, EPS_83124_ and EPS_83123_ presented modifications in monosaccharide composition, with a reduction of rhamnose and an increase of amino-sugars as temperature rise. These changes in the structure of EPS_83124_ resulted in an increase of the apparent viscosity of milks fermented at 20°C (223 mPa.s) and 30°C (217 mPa.s) with respect to acid gels obtained at 37°C (167 mPa.s). In order to deepen the knowledge on EPS characteristics, monosaccharide composition of low and high molecular weight EPS fractions were evaluated. Finally, it was evidenced that the preincubation of intestinal epithelial cells Caco-2/TC-7 with EPS_8339_ and EPS_83124_ partially inhibit the association and invasion of *Salmonella*. In light of these results, it can be concluded that the selection of the EPS-producing strain along with the appropriate fermentation conditions could be an interesting strategy to improve the technological properties of these *L. paracasei* fermented milks with potential protective effects against intestinal pathogens.

## 1. Introduction

The selection of lactic acid bacteria (LAB) for dairy manufacturing includes the search for exopolysaccharides-producing strains to fulfill consumer demand of pleasant and healthy products without additives. Exopolysaccharides are produced *in situ* during fermentation, having a great impact on the physico-chemical characteristics of the final product and contributing to enhance sensorial attributes on account of their techno-functional properties (Lynch et al., 2018; Dedhia et al., 2022). These biopolymers can act as bio-thickeners improving rheological properties such as viscosity of fermented beverages or viscoelastic properties of acid milk gels (Rimada and Abraham, 2006; Xu et al., 2019). Additionally, they can prevent syneresis due to their water holding capacity and may act as emulsion stabilizing agents (Torino et al., 2015; Piermaria et al., 2021). Exopolysaccharides produced by LAB can be secreted into the growth media and/or form a slime layer loosely bound to the bacterial surface (EPS) or remain tightly linked to the cell surface forming a capsular polysaccharide (CPS) (Ruas-Madiedo et al., 2009; Oleksy and Klewicka, 2018; Bachtarzi et al., 2020). This extracellular location of EPS allows the interaction of these polymers with the matrix improving the physico-chemical properties of products obtained by fermentation with EPS-producing strains.

Some LAB strains with demonstrated probiotic properties are able to produce EPS. In fact, surface EPS plays a key role in the interaction of the probiotic bacteria with epithelial cells, triggering the biological effect (Bengoa et al., 2018a; Sabater et al., 2020). In fact, the health-promoting effect attributed to the probiotic bacteria has been ascribed to the presence of these biopolymers in many cases (Ale et al., 2020; Bengoa et al., 2021; Werning et al., 2022). Among the diverse health promoting properties ascribed to EPS produced by LAB it can be mentioned their ability to modulate intestinal microbiota, exert immunomodulatory and/or antitumor effect, or reduce cholesterol level (Laiño et al., 2016; Ale et al., 2020; Medrano et al., 2020; Bengoa et al., 2020a; Oerlemans et al., 2021; Simonelli et al., 2022).

It has been previously demonstrated that there is a direct relationship between EPS structure and its functionality, both technological and biological (Welman and Maddox, 2003; Chen et al., 2019; Bengoa et al., 2020a). In this direction, EPS-producing LAB are consider special suppliers of a variety of novel polysaccharides whose monomer composition, anomeric configuration, glycosidic linkage and structure is defined by specific biosynthesis enzymes present in each microorganism, resulting in an enormous EPS diversity with potentially different applications (Zeidan et al., 2017).

Since remotes times, fermented foods have been widely produced and consumed as part of a healthy diet, not only because of their enhanced food safety and extended shelf-life but also due to their human health benefits associated (Leeuwendaal et al., 2022). Recently, scientific attention was focused on artisanal fermented foods such as kefir, sugary kefir, kimchi, among others as sources of novel LAB strains with promising technological properties and probiotic potential (Tamang et al., 2016; Bengoa et al., 2019b; Pendón et al., 2021). Isolation of novel strains from these fermented foods would increase microbial biodiversity in the development of selected starter cultures with defined properties (Bachtarzi et al., 2020; Galli et al., 2022). Moreover, they can be the source of novel EPS to fulfill expectation of technological progress for their application in dairy foods (Bachtarzi et al., 2019), non-dairy food (Llamas-Arriba et al., 2019), or biotherapeutic products (de Carvalho and Conte-Junior, 2021; Nadzir et al., 2021).

In previous reports, the isolation of several EPS-producing strains from kefir grains was described by our group (Hamet et al., 2013, 2015; Gangoiti et al., 2017). Among them, *Lacticaseibacillus paracasei* CIDCA 8339, CIDCA 83123 and CIDCA 83124 have proven to be promising probiotic strains that have the ability to adhere to gastric and intestinal epithelial cells even after the passage through the gastrointestinal tract (Zavala et al., 2016; Bengoa et al., 2018b, 2020b) and also meet the requirements established by the regulations about safety for food application (Bengoa et al., 2019a).

One of the effectors ascribed to health-promoting properties of *L. paracasei* strains are exopolysaccharides (Bengoa et al., 2021). In particular, exopolysaccharide produced in milk by *L. paracasei* CIDCA 8339 and CIDCA 83124 strains have demonstrated their potential to modulate the fecal microbiota of children *in vitro* inducing changes in microbial populations and increasing the production of propionic and/or butyric acid depending on the EPS used (Bengoa et al., 2020a). Additionally, it was evidenced that EPS-production by these strains on MRS broth is dependent on incubation temperature with an increment of high molecular weight fractions at low temperature, along with an increase in the total amount of EPS produced (Bengoa et al., 2018a).

Taking in consideration the previous results in MRS medium and the biological effect of EPS produced in milk by these strains, the aim of the present work is to evaluate the effect of fermentation temperature on the characteristics of EPS produced by *L. paracasei* strains in milk and the consequent impact on the rheological and functional properties of the fermented products.

## 2. Materials and methods

### 2.1. Bacterial strains and culture conditions

Stock cultures of EPS-producing *Lacticaseibacillus paracasei* CIDCA 8339, CIDCA 83123 and CIDCA 83124 isolated from kefir grains were stored in 12% w/v non-fat milk solids at −80°C. Strains were grown in MRS broth (Difco Laboratories, Detroit, MI, USA) at 20°C (48 h), 30°C (24 h) and 37°C (24 h) under aerobic conditions previous to each experiments (Bengoa et al., 2018a). *Salmonella enterica* serovar Enteritidis CIDCA 101 (Zavala et al., 2016) used for association/invasion experiments was grown in nutrient broth (Biokar Diagnostics, Beauvais, France) for 18 h at 37°C.

### 2.2. Fermented milks preparation and characterization

Fresh pure cultures of each strain were inoculated in 10 mL of ultra-high temperature low fat milk (La Serenisima, Mastellone Hnos S.A, Argentina) at 5% v/v and incubated at 20°C, 30°C or 37°C. pH and viable bacteria were evaluated at different fermentation times until pH was lower than 4.0. The number of viable bacteria in the fermented milks (CFU/mL) was determined in MRS agar plates. Temperatures selected were between the range of temperature previously described for this species (Zheng et al., 2020).

Organic acids levels in the fermented milks were determined qualitatively and quantitatively by high pressure liquid chromatography (HPLC) when the culture reached pH ≤ 4.0 according to Bengoa et al. (2019a). Briefly, 1 mL of the fermented product was centrifuged for 10 min at 10,000 × *g*. The supernatant obtained was filtered through a 0.45 μm membrane (Millipore Corporation, USA) and 20 μL of the filtrate were injected into the chromatograph. Organic acids were separated on an AMINEX HPX-87H ion exchange column (BioRad Labs, USA) and detected at 214 nm (Waters ™ 996, Millipore Corporation, Milford, MA 01757, USA). The determination was carried out at a flow rate of 0.7 mL/min at 60°C using 0.009 N H_2_SO_4_ as mobile phase for 30 min. Acids were identified by comparison of the retention times with HPLC-grade standard solutions (Sigma Chemical Co, USA). Additionally, calibration curves of lactic and acetic acid standards were used to determine the concentration of these acids in the samples.

Flow behavior at 25°C of fermented milks was determined in a Haake ReoStress 600 rheometer (Haake, Karlsruhe, Germany) in its rotational mode using a 1 mm gap plate-rough plate sensor system PP35 (Thermo Haake, Karlsruhe, Germany) according to Hamet et al. (2015). The samples were subjected to a cycle that consisted of an increase of shear rate up to 500 s^−1^ using an acceleration of 4,167 s^−2^. Then, the same but negative acceleration value was used to return the shear rate to 0 s^−1^. Flow curves were obtained and the apparent viscosity at 300 s^−1^ (mPa.s) was determined for each sample. The flow behavior of fermented milks adjusted with Ostwald de Waele model: *τ* = *k.*𝛾 ˙^n^, where *τ* is the shear stress (Pa), *k* is the consistency index (Pa s*^n^*), 𝛾̇ is the shear rate (s^−1^) and n is the flow index (non-dimensional); or Carreau-Yasuda model: *τ =* 𝛾̇ [*η*_∞_ + (*η*_0_ − *η*_∞_) / (1 + (λ 𝛾̇) *^a^*) ^(1-n)/*a*^], where *τ* is the shear stress (Pa), 𝛾̇ is the shear rate (s^−1^), *η*_0_ is the initial viscosity expressed in Pa·s, *η*_∞_ is the viscosity to infinite time expressed in Pa·s and its constant value is 0, n is the flow behavior index, *λ* is time parameters in s^−1^ and *a* is a constant (non-dimensional). Glucono δ lactone (GDL) acid milk gels were obtained according to Rimada and Abraham (2006) and used as controls.

### 2.3. EPS extraction

For EPS extraction, 500 mL of commercial UHT low fat milk (La Serenisima, Mastellone Hnos S.A, Argentina) were individually inoculated with fresh pure cultures (1% v/v) and incubated at 20°C, 30°C or 37°C until the acid gels were formed. The fermented milks obtained were heated for 30 min at 100°C in order to favor the detachment and dissolution of the polysaccharide bound to the cells and allow enzymes denaturalization. Trichloroacetic acid 8% w/v (Ciccarelli, Argentina) was added to precipitate the proteins. Then, the samples were centrifuged at 10,000 *g* for 20 min at 20°C in an Avanti J25 centrifuge (Beckman Coulter Inc., USA) and two volumes of cold ethanol were added to the supernatant obtained. The samples were placed at-20°C overnight and centrifuged at 10,000 *g* for 20 min at 4°C. EPS pellets were dissolved in hot distilled water and dialyzed against bi-distilled water through a 1 kDa cut-off dialysis membrane (Spectra/Por, The Spectrum Companies, Gardena, CA) for 48 h at 4°C. Finally, the samples were lyophilized. EPS extraction was performed from two independent cultures. The absence of other sugars was determined by thin-layer chromatography and the absence of proteins was evaluated by the Bradford method (Rimada and Abraham, 2003). EPS amount was estimated by weight of crude EPS lyophilized and expressed as mg/L.

### 2.4. Evaluation of molecular weight distribution and monosaccharide composition of EPS

Molecular weight distributions of lyophilized crude EPS were determined by size exclusion chromatography. In brief, crude EPS powder was suspended in 0.1 M NaNO_3_ (0.5 mg/mL) and then filtered through a 0.45 μm pore diameter polyvinylidene fluoride membrane (Millipore Corporation, USA). The average molecular weight (MW) was determined by high-performance molecular exclusion chromatography (HPLC-SEC, Agilent 1,100 Series System, Hewlett-Packard, Germany) associated with a refractive index (IR) detector (Ibarburu et al., 2015). 50 μL of the samples were injected and eluted at a flow rate of 0.95 mL/min (pressure: 120:130 psi) at room temperature using 0.1 M NaNO_3_ as mobile phase. Dextrans (0.5 mg/mL) with a molecular weight between 10^3^ and 2.10^6^ Da (Sigma-Aldrich, USA) were used as standards.

Once the molecular weight distributions were determined, low and high molecular weight fractions that composed the crude EPS obtained at 20°C were separated. For this purpose, EPS solutions (0.2% w/v) were centrifuged through a Vivaspin™ ultrafiltration spin column 100 KDa MWCO, (Sartorious, Goettingen, Germany) for 20 min at 6000 *g*, eluting only the low MW fraction. Subsequently, high MW fraction retained in the column was eluted using hot distilled water. The eluted fractions were passed through a Vivaspin column (cut-off 30KDa) in order to separate the middle and low MW fraction of EPS.

Monosaccharide composition of crude EPS and their fractions were determined by gas chromatography as previously described (Notararigo et al., 2013). Briefly, 1–2 mg of EPS were hydrolyzed in 1 mL of 3 M trifluoroacetic acid (1 h at 120°C). The monosaccharides obtained were converted into alditol acetates by reduction with NaBH_4_ and subsequent acetylation. The samples were analyzed by gas chromatography in an Agilent 7890A coupled to a 5975C mass detector, using an HP5-MS column with helium as carrier gas at a flow rate of 1 mL/min. For each run, 1 μL of sample was injected (with a Split 1:50) and the following temperature program was performed: the oven was heat to 175°C for 1 min; the temperature was increased to 215°C at a rate of 2.5°C/min and then increased to 225°C at 10°C/min, keeping it constant at this temperature for 1.5 min. Monosaccharides were identified by comparison of retention times with standards (arabinose, xylose, rhamnose, galactose, glucose, mannose, glucosamine and galactosamine) analyzed under the same conditions. Calibration curves were also processed for monosaccharide quantification. Myo-inositol was added to each sample as internal standard.

### 2.5. *Salmonella* association and invasion assays

*Salmonella* association and invasion assays were performed according to Zavala et al. (2016). Caco-2/TC-7 cells that were routinely grown in Dulbecco’s modified Eagle’s minimal essential medium (DMEM) (GIBCO BRL Life Technologies Rockville, MD. USA), supplemented with 15% heat-inactivated (30 min at 60°C) fetal bovine serum (FBS, PAA, GE Healthcare Bio-Sciences Corp., USA), 1% non-essential amino acids (GIBCO BRL Life Technologies Rockville, MD. USA), and the following antibiotics (Parafarm, Saporiti SACIFIA, Buenos Aires, Argentina): penicillin (12 UI/mL), streptomycin (12 μg/mL), gentamicin (50 μg/mL). Cells were seeded in 24-well culture plates (Corning, NY, USA) at 2.5 × 10^5^cells per well and incubated at 37°C in a 5% CO_2_ — 95% air atmosphere. Caco-2/TC-7 cells were used at post-confluence after 7 days of culture.

*Salmonella enteritidis* serovar enteritidis CIDCA 101, provided by Dr. H. Lopardo, was grown in nutritive broth (Biokar Diagnostics, Beauvais, France) for 18 h at 37°C (Golowczyc et al., 2007). Confluent Caco-2/TC-7 monolayers were washed twice with sterile PBS (pH 7.2). Cells were pre incubated for 1 h at 37°C in a 5% CO_2_—95% air atmosphere with 250 μL EPS solutions (300, 500 and 800 mg/L in DMEM) or 250 μL DMEM in the case of *Salmonella* association and invasion controls. Afterwards, 250 μL of *Salmonella* suspension (1 × 10^7^ CFU/mL) were added to each well and incubated 1 h at 37°C in a 5% CO_2_—95% air atmosphere. For association assays, cells were washed three times with PBS and lysed with 500 μL/well of bi-destilled water. The number of associated *Salmonella* (adhering and invading) was determined by serial dilutions on 0.1% w/v tryptone followed by colony counts on nutrient agar. *Salmonella* invasion was determined by counting only bacteria located in the Caco-2/TC-7 cells. For this purpose, the monolayer incubated with *Salmonella* as previously described, were treated with 0.5 mL/well of gentamicin (100 μg/mL PBS) for 1 h at 37°C. Subsequently, cells were lysed and colony counts performed as described above.

### 2.6. Statistical analysis

All experiments were performed at least in triplicate. Quantitative data were analyzed by using one-way analysis of variance (ANOVA) followed by Tukey’s Tests for multiple mean comparisons. A value of *p* <0.05 indicates significant differences. GraphPad Prism version 5.01 software for Windows (GraphPad®, California, USA) was used for data analysis. The results are expressed as mean ± standard deviation (SD).

## 3. Results

### 3.1. Milk fermentation with *Lacticaseibacillus paracasei* strains at different temperatures

**Figure 1:**
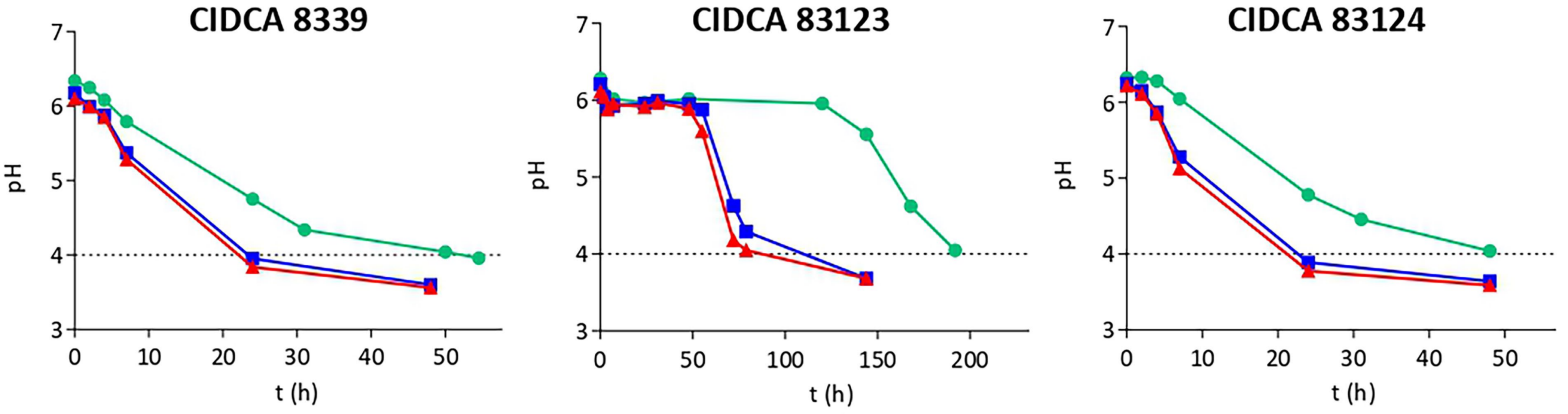
Acidification kinetics of *Lacticaseibacillus paracasei* CIDCA 8339, CIDCA 83123 and CIDCA 83124 in milk at 20°C (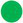), 30°C (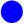), and 37°C (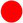). Dotted line indicates the pH selected for characterization of fermented milks.

As a first characterization, the growth and acidification kinetics of *L. paracasei* strains in milk at 20, 30 and 37°C were evaluated (Table 1; Figure 1) and the fermented milks obtained were characterized. Table 1 shows the growth rate and yield of the strains, and organic acids concentration in all cases. It was clearly evidenced that growth and acidification rate were influenced by incubation temperature, observing an increase with temperature for the three strains without showing significant differences in the growth yield. *L. paracasei* CIDCA 8339 and CIDCA 83124 showed an acceptable growth and acidification rate even at 20°C, requiring between 24 h at 30 and 37°C, and 48 h at 20°C to reach the maximum growth and a pH ≤ 4.0. On the other hand, *L. paracasei* CIDCA 83123 showed low growth and acidification rates at all temperatures assayed, demanding a longer fermentation period to form the acid gels and reach the maximum growth. In fact, the pH of the milk fermented with *L. paracasei* CIDCA 83123 at 20°C began to decrease only after 125 h of fermentation, requiring 192 h (8 days) to obtain the acid gel at this temperature. These differences in growth and acidification kinetics between *L. paracasei* strains at 20°C can be clearly evidenced in Supplementary Figure S1. The slow growth of CIDCA 83123 in milk constitutes a technological disadvantage for this strain since the food industry usually prefers the application of fast-growing acidifying microorganisms for the development of fermented dairy products.

**Table 1:** Bacteria growth rate and yield, lactic and acetic acid concentration of milk fermented with *Lacticaseibacillus paracasei* CIDCA 8339, CIDCA 83123, and CIDCA 83124 at 20°C, 30°C, and 37°C.

*L. paracasei* strain	Temperature	Growth rate (h^−1^)	Growth yield (log CFU/mL)	Lactic acid (mM)	Acetic acid (mM)
CIDCA 8339	20°C	0.0718	9.45 ± 0.08	142.54 ± 15.02 ^a^	9.05 ± 1.85 ^a^
	30°C	0.1088	9.38 ± 0.05	150.15 ± 11.07 ^a^	12.38 ± 4.94 ^ac^
	37°C	0.1645	9.28 ± 0.11	143.57 ± 11.77 ^a^	7.85 ± 1.12 ^a^
CIDCA 83123	20°C	0.0055	9.68 ± 0.08	126.58 ± 7.77 ^a^	20.66 ± 1.29 ^b^
	30°C	0.0138	9.63 ± 0.02	124.72 ± 26.08 ^a^	21.43 ± 0.53 ^b^
	37°C	0.0178	9.79 ± 0.09	122.78 ± 4.61 ^a^	19.46 ± 3.59 ^bc^
CIDCA 83124	20°C	0.0556	9.75 ± 0.11	119.55 ± 9.61 ^a^	8.62 ± 2.11 ^a^
	30°C	0.1703	9.80 ± 0.08	132.76 ± 24.39 ^a^	10.23 ± 1.56 ^a^
	37°C	0.2387	9.79 ± 0.04	154.64 ± 28.64 ^a^	11.52 ± 1.27 ^a^

Different letters indicate significant differences (*p* < 0.05).

Subsequently, the fermented products obtained at different temperatures were characterized with regard to macroscopic aspect, pH and organic acids levels. The fermented products obtained with the strains at all three temperatures were firm acid gels with pleasant appearance that did not presented syneresis. Those obtained with CIDCA 8339 and CIDCA 83123 strains did not show a ropy character. However, milks fermented with CIDCA 83124, at 20°C and 30°C showed a marked ropy character (Supplementary Figure S2) that was not evidenced when milk was fermented at 37°C.

Moreover, it could be evidenced that lactic acid levels (120–150 mM) in the milks fermented with different *L. paracasei* strain did not present significant differences (Table 1). On the other hand, *L. paracasei* CIDCA 83123 produced higher levels of acetic acid than CIDCA 8339 and CIDCA 83124 strains. However, for a specific strain, no significant differences were observed in lactic or acetic acid levels with fermentation temperature.

The results obtained indicate that although temperature incubation has a significant impact on the growth and acidification kinetics of *L. paracasei* strains, at the end of fermentation organic acid levels, pH and probiotic concentration result equivalent. At this point, *L. paracasei* CIDCA 8339 produces 130–145 mg of EPS per liter of fermented milk obtained at 30°C, CIDCA 83123 produces 155–160 mg/L and CIDCA 83124 about 140–160 mg/L, being these values not significantly different.

**Figure 2:**
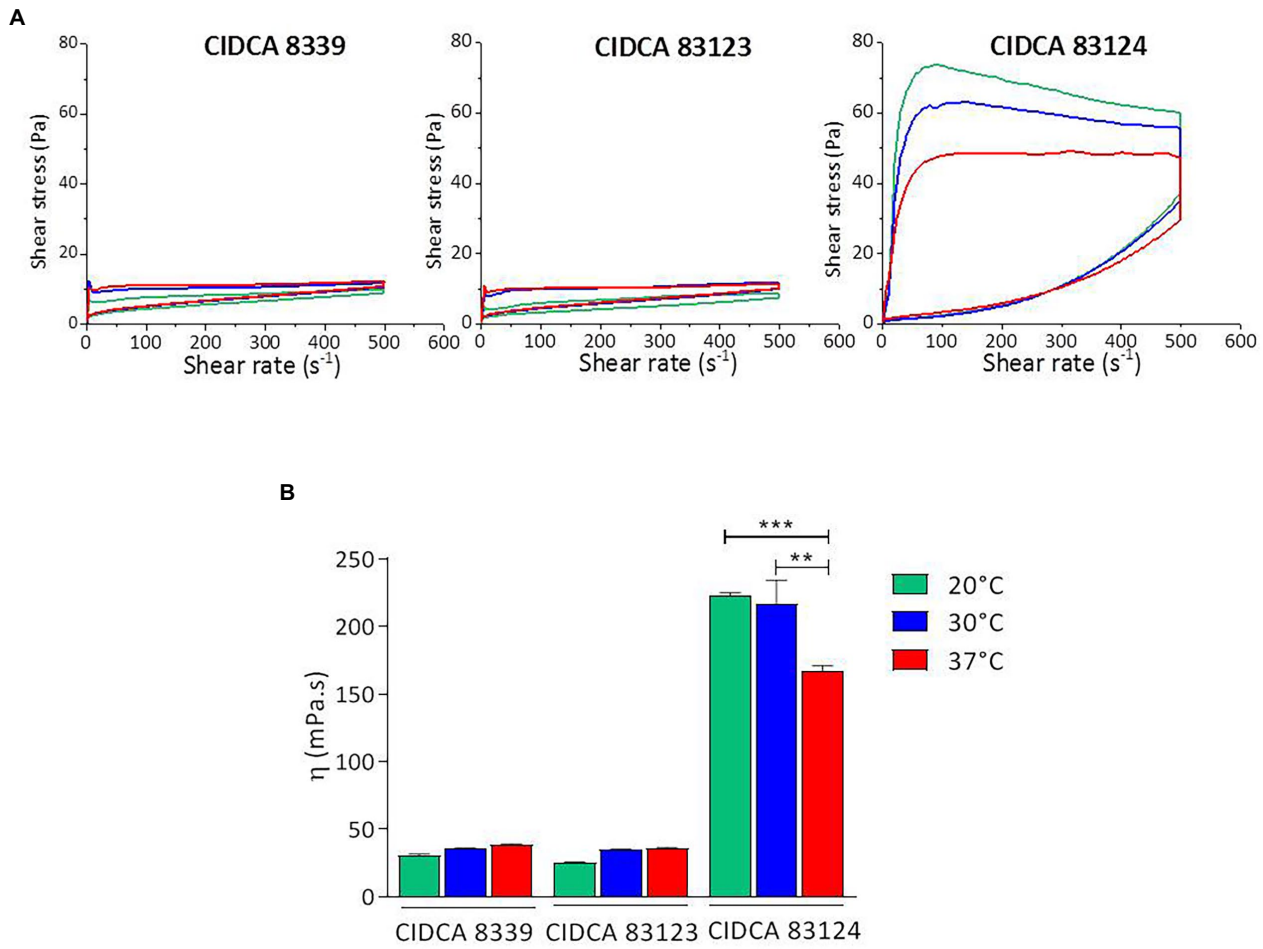
Flow curves **(A)** and apparent viscosity at 300 s^−1^ **(B)** of milks fermented with *L. paracasei* CIDCA 8339, CIDCA 83123, and CIDCA 83124 at 20°C (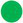), 30°C (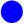), and 37°C (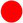). Significant differences ** *p* < 0.01, *** *p* < 0.001.

Flow behavior and apparent viscosity of the fermented products were analyzed (Figure 2). All acid gels obtained presented a non-Newtonian pseudoplastic behavior with a hysteresis loop that was higher in the milks fermented with CIDCA 83124 at all temperatures (Figure 2A). The hysteresis is a measure of the extent of structural breakdown during the shearing cycle. The evidence of this structural breakdown was demonstrated by analyzing the flow behavior in the up and down curve. Fermented milk with *L. paracasei* CIDCA 8339 at 20°C and CIDCA 83123 at 20°C or 30°C presented a flow behavior that fitted the Ostwald de Waele model with n values ranging from 0.08 to 0.2 in the up curve while fermented milks with *L. paracasei* CIDCA 8339 at 30°C and 37°C or *L. paracasei* CIDCA 83123 at 37°C did not fit this model (Supplementary Table S1). However, after the first structural breakdown (flow behavior of the down curve) all the acid gels obtained with these strains fitted Ostwald de Waele model with n values between 0.41 and 0.47. On the other hand, all the acid gels obtained by fermentation with CIDCA 83124 did not fit Otswald de Waele model indicating that these acid gels have a different texture, may be due to differences in the characteristic of EPS produced by this strain. These acid gels fitted to Carreau-Yasuda model (Supplementary Table S2) which describes a pseudoplastic fluid with asymptotic viscosities at zero (η_0_) and infinite (*η*_∞_) shear rates with no yield stress (Hackley and Ferraris, 2001).

Moreover, the acid gels obtained by fermentation with CIDCA 83124 were significantly more viscous, compared to those fermented by *L. paracasei* CIDCA 8339 and CIDCA 83123 (Figure 2B; Supplementary Table S3). Regarding the effect of fermentation temperature, significant changes in the apparent viscosity of the fermented milk were only observed with *L. paracasei* CIDCA 83124, obtaining values significantly higher at 20°C and 30°C. These results indicate that the fermentation temperature could be modifying the physico-chemical characteristics of the EPS synthesized by this strain.

Flow curve of milk gel acidified with GDL used as control fitted Otswald de Waele model having a lower consistence index and a flow index (n) of 0.8 (Supplementary Table S2). The smaller value of n of acid gels obtained by milk fermentation with *L paracasei*, may indicate that EPS affects structure of the acid gels and consequently modify their flow behavior.

### 3.2. Temperature fermentation modified the molecular weight of exopolysaccharides produced by *Lacticaseibacillus paracasei* strains

Crude EPS synthesized by the strains at 20, 30 and 37°C in milk were extracted and subsequently characterized in terms of molecular weight distribution (MW). Table 2 shows the molecular weight fractions evidenced for the EPS produced at different temperatures and the percentage relative area of the MW fractions in each condition. *Lacticaseibacillus paracasei* CIDCA 8339 synthesizes an EPS that is made up of two fractions, a high MW fraction in the order of 10^5^ Da and a low MW fraction of about 10^4^ Da. These two fractions are present at the three temperatures studied, observing a change in the relative abundance of each fraction depending on the temperature. The low MW fraction is the most abundant when fermentation temperature is 37°C. At 30°C, the proportion of both fractions is equivalent; meanwhile the high MW fraction becomes predominant at 20°C. The polysaccharide produced by CIDCA 83124 at 20 and 37°C is composed by two fractions, a low MW fraction of 10^4^ Da and a high MW of 1–4 × 10^6^ Da. This last fraction has a higher MW than the corresponding produced by CIDCA 8339. The EPS produced by *L. paracasei* CIDCA 83124 at 30°C presents two additional fractions that include an intermediate MW fraction of 7 × 10^4^ Da and another high MW fraction of 7 × 10^5^, being composed by a total of four fractions. When analyzing the EPS produced by *L. paracasei* CIDCA 83123, it can be evidenced that there are four distributions of different MW at all three temperatures. As evidenced with CIDCA 8339, a clear increase in the proportion of the high MW fractions at lower fermentation temperatures was also observed for CIDCA 83123 and CIDCA 83124 when grown in milk.

**Table 2:** Molecular weight and relative abundance (percentual expression) of EPS fractions produced by *L paracasei* strains in milk at different temperatures.

*L. paracasei* strain	Temperature	EPS molecular weight (Da)							
		High MW fraction (>1 × 10^5^)				Middle MW fraction (3 × 10^4^ < PM > 1 × 10^5^)		Low MW fraction (<3 × 10^4^)	
		Mw	Relative abundance %	Mw	Relative abundance %	Mw	Relative abundance %	Mw	Relative abundance %
CIDCA 8339	20°C	-	-	1.4 × 10^5^	57.1	-	-	9.0 × 10^3^	42.9
	30°C	-	-	3.9 × 10^5^	50.0	-	-	1.0 × 10^4^	50.0
	37°C	-	-	5.4 × 10^5^	33.3	-	-	1.2 × 10^4^	66.7
CIDCA 83123	20°C	3.4 × 10^6^	85.1	6.0 × 10^5^	6.4	7.4 × 10^4^	2,1	1.0 × 10^4^	6.8
	30°C	3.3 × 10^6^	17.6	6.8 × 10^5^	17.6	7.1 × 10^4^	5.9	1.1 × 10^4^	58.9
	37°C	3.5 × 10^6^	9.5	4.5 × 10^5^	14.3	7.8 × 10^4^	9.5	1.2 × 10^4^	66.7
CIDCA 83124	20°C	3.7 × 10^6^	80.0	-	-	-	-	1.1 × 10^4^	20.0
	30°C	5.7 × 10^6^	21.0	6.9 × 10^5^	10.5	7.0 × 10^4^	5.3	1.1 × 10^4^	63.1
	37°C	1.1 × 10^6^	14.3	-	-	-	-	1.5 × 10^4^	85.7

Relative abundance is calculated considering the percentage of the area of each peak referred to the total area of the chromatogram. Standard deviation ranged between 0.05 and 0.2. Standard deviation in retention time of peak ranged from 0.04 to 0.06.

### 3.3. Monosaccharide composition of exopolysaccharide produced by *Lacticaseibacillus paracasei* was affected by temperature fermentation depending on the strain

**Figure 3:**
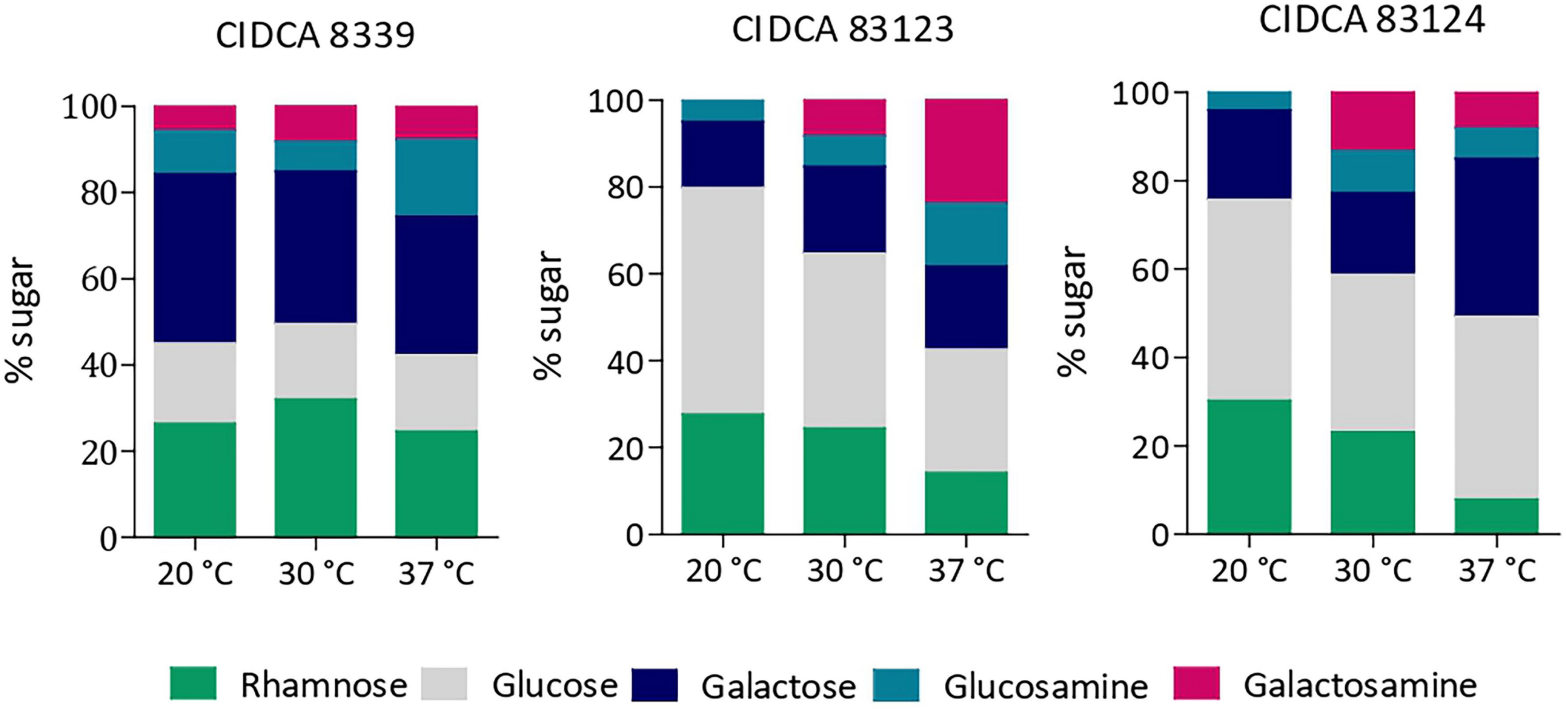
Monosaccharide composition of EPS produced by *L. paracasei* CIDCA 8339, CIDCA 83123 and CIDCA 83124 in milk at 20°C, 30°C, and 37°C. Standard deviation in monosaccharide composition was between 0.2 and 4 depending on the percentage of each monosaccharide.

Monosaccharide composition of the crude EPS produced in milk by the three *L. paracasei* strains was evaluated, evidencing that all of them contain glucose, galactose and rhamnose as major sugars, and to a lesser extent amino sugars such as glucosamine and galactosamine. However, the relative amount of each monosaccharide depended on the strain and growth temperature (Figure 3).

EPS from *L. paracasei* CIDCA 8339 is mainly composed of galactose and rhamnose and to a lesser extent glucose, keeping the proportion of sugars constant at the three temperatures studied. On the contrary, EPS from CIDCA 83123 and CIDCA 83124 strains are mainly composed by glucose. Moreover, changes in the proportion of sugar composition with temperature were evidenced for these two stains. EPS produced by CIDCA 83123 at 20°C has glucose as the most abundant sugar, followed by galactose and rhamnose. However, the proportion of glucose and rhamnose decreases when temperature fermentation increases, accompanied by a rise in glucosamine and galactosamine proportion. Similarly, EPS of *L. paracasei* CIDCA 83124 also showed a reduction in rhamnose at higher temperatures, along with an increase in galactose and amino sugars. In summary, it can be state that in the case of EPS produced by CIDCA 83123 and CIDCA 83124 the incorporation of rhamnose is favored at 20°C, while the proportion of amino sugars increases at higher temperatures.

**Figure 4:**
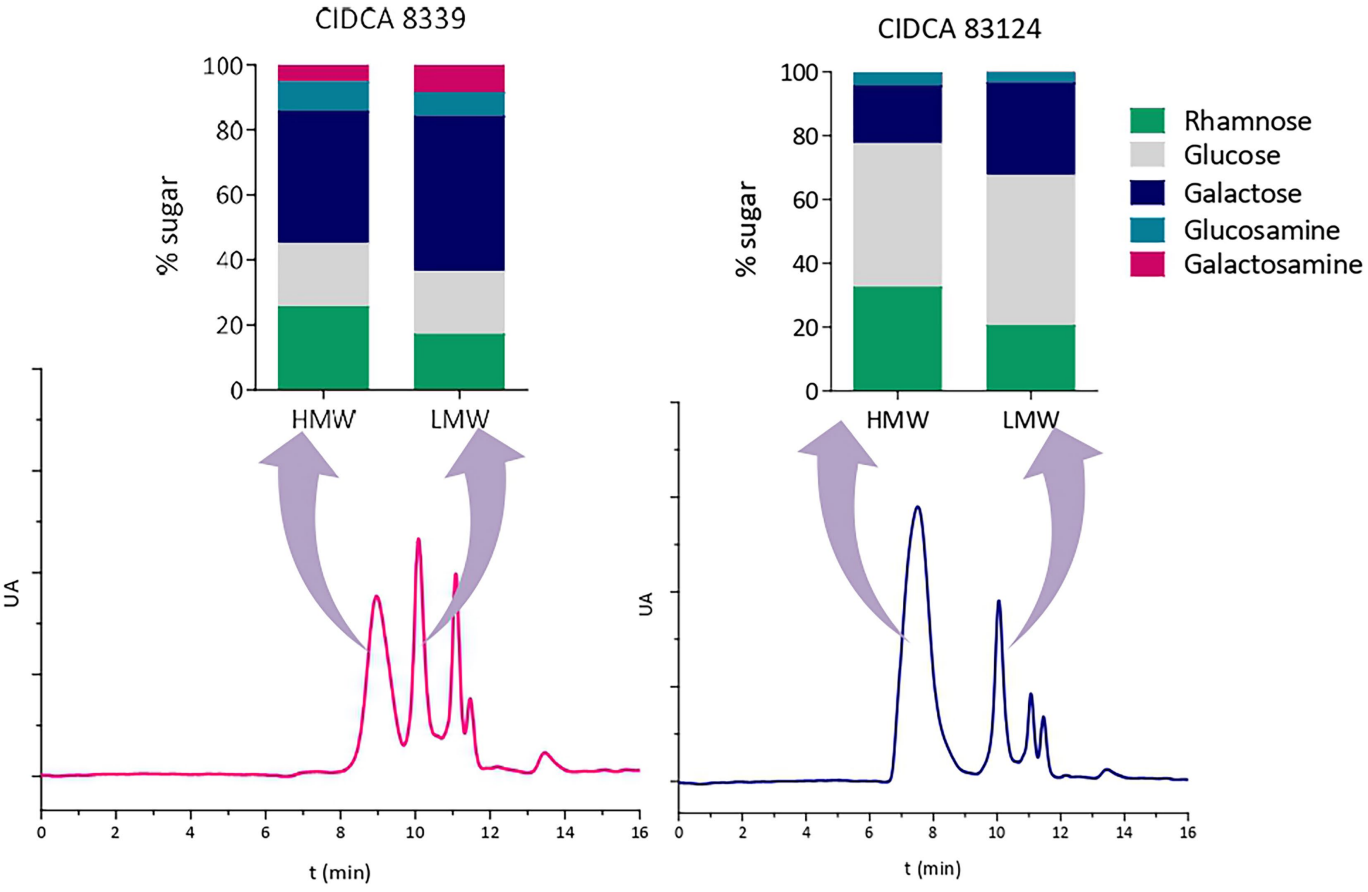
High-performance size exclusion chromatograms of EPS isolated from milk produced by *L. paracasei* CIDCA 8339 and CIDCA 83124 at 20°C, and monosaccharide composition of the corresponding high and low molecular weight fractions. AU: arbitrary units. Standard deviation in monosaccharide composition was between 0.4 and 4 depending on the percentage of each monosaccharide.

Then, monosaccharide compositions of high and low molecular weight fractions of EPS were studied (Figure 4). For this purpose, EPS produced at 20°C were selected since high and low molecular weight fractions were clearly defined in the chromatograms, facilitating their separation and purification. At 20°C the two fractions of EPS produced by *L. paracasei* CIDCA 8339 (EPS_8339_) presented similar monosaccharide composition showing only small differences in the proportion of galactose and rhamnose. Similar results were observed in the case of EPS_83124._. The similarity in monosaccharide composition of low and high molecular weight fraction of these EPS could be indicating that both fractions correspond to the same polymer but with different polymerization grade.

### 3.4. EPS from *Lacticaseibacillus paracasei* CIDCA 8339 and CIDCA 83124 protected against *Salmonella* invasion *in vitro*

The protective effect of EPS against *Salmonella enteritidis* infection to Caco-2/TC-7 cells was evaluated. Considering all previous results and in sight of applying these strains for the development of a fermented product, EPS produced by CIDCA 8339 and CIDCA 83124 at 30°C were selected since these strains presented an adequate fermentation time to reach pH <4.0 at this temperature. Moreover, the comparison of these two EPS results particularly interesting due to the differences evidenced in crude EPS molecular weight distribution and monosaccharide composition which could imply differences in the biological activity.

To evaluate the protective effect of EPS_8339_ and EPS_83124_, cells were pre-incubated with EPS solutions before *Salmonella* infection. As shown in Table 3, when the concentration was 800 mg/L, the presence of EPS_8339_ and EPS_83124_ led to a reduction of 0.5 log of the pathogenic bacteria associated to Caco-2/TC-7 cells when compared to the control. Moreover, the internalization of *Salmonella* strain to these intestinal epithelial cells was affected by EPS observing a significant decrease of 1.5 log in the number of *Salmonella* internalized to the cells. Nevertheless, lower concentrations of EPS (300 or 500 mg/L) did not exert a protective effect. These results could be explained by a barrier effect ejected by EPS that prevents the interaction of the bacteria with specific cell receptors hindering the adhesion and invasion of *Salmonella* and avoiding the setting up of the infection.

**Table 3:** Association and invasion of *Salmonella enteritidis* to Caco-2/TC-7 cells preincubated (1 h, 37°C) with EPS produced by *L. paracasei* CIDCA 8339 and CIDCA 83124 at 30°C in milk.

	Concentration (mg/L)	Association (log CFU/mL)	Invasion (log CFU/mL)
Control	-	6.07 ± 0.06^a^	4.60 ± 0.05^a^
EPS 8339	300	6.07 ± 0.01^a^	4.69 ± 0.09^a^
	500	6.14 ± 0.03^a^	4.57 ± 0.08^a^
	800	5.49 ± 0.04^b^	3.50 ± 0.04^b^
EPS 83124	300	6.03 ± 0.12^a^	4.63 ± 0.04^a^
	500	6.08 ± 0.02^a^	4.55 ± 0.11^a^
	800	5.54 ± 0.03^b^	3.43 ± 0.08^b^

Control corresponds to cells preincubated with DMEM. Different letters within columns indicate significant differences (*p* < 0.05, Tukey’s multiple comparison test).

## 4. Discussion

In the present research the growth in milk at different temperatures of three EPS-producing *L. paracasei s*trains isolated from kefir was evaluated focusing the study on the characterization of EPS. The response of microorganisms to environmental conditions has been extensively investigated. Particularly for LAB, the physiological and molecular mechanisms in response to growth temperature have been studied due to the impacts on food processing (De Angelis and Gobbetti, 2004). According to König and Berkelmann-Löhnertz (2017) and Sánchez et al. (2019), in general the optimal growth temperature of lactobacilli is between 30 to 40°C; however, there are strains that can grow at temperatures ranging from 2 at 53°C as was observed for *L. plantarum* strains that were able to grow at low temperatures (4°C to 16°C) (Dalcanton et al., 2018). Likewise, other authors have found that some *Lactobacillus* spp. can grow at high temperatures (Yang et al., 2018; Śliżewska and Chlebicz-Wójcik, 2020). The effect of temperature on LAB growth has also been studied in different food matrices such as fruit juice (Mustafa et al., 2019) or coconut milk (Saori Ishii Mauro and Garcia, 2019). When evaluating the growth of different strains of LAB in fruit juice at 30, 35 and 37°C, a strain-dependent effect was observed since some strains had an equivalent growth at different temperatures while others showed better development at 30°C. However, none of the strains presented marked changes in lactic acid production with temperature (Mustafa et al., 2019). This is in concordance with our results where temperature affected growth rate in a strain specific manner. When studying the effect of temperature on milk fermentation, it has been evidenced that the production of metabolites by probiotic strains occurs in different amounts depending on the temperature and time of fermentation, which illustrates the relevance of controlling these parameters (Østlie et al., 2005). Organic acids constitute one of the main metabolites produced by LAB during milk fermentation. In the present work, it was evidenced an influence of temperature in acidification kinetics. However, as organic acids were determined at the end of the fermentation process which was defined by a pH ≤ 4.0, the concentration of viable microorganisms as well as organic acids concentrations showed no significant differences with temperature fermentation.

As previously mentioned, some LAB are able to produce exopolysaccharides, which are relevant metabolites in food due to their potential biological and techno-functional properties. To deepen the knowledge about the characteristics of EPS produced by *L. paracasei* CIDCA strains and understand the relationship between EPS features and their functional properties, the degree of polymerization and the monomer composition of each EPS were studied.

EPS produced by LAB present a wide range of molecular weight that varies between 10^4^ and 10^6^ Da for heteropolysaccharides (HePS) and up to 10^8^ for homopolysaccharides (HoPS) (Ryan et al., 2015; Daba et al., 2021; Werning et al., 2022), as it was evidenced for *L. paracasei* strains reported in the literature (Liu et al., 2013; Wang et al., 2022). When studying the molecular weight of EPS from *L. paracasei* CIDCA strains, it was observed that these EPS have fractions with different molecular weight that goes from low MW fractions of 1 × 10^4^ Da to high MW fractions of 5.10^6^ Da. It has been described that some LAB produce EPS fractions that could be ascribed to different EPS (Ibarburu et al., 2015; Llamas-Arriba et al., 2019) while others usually produce fractions that only differ in the degree of polymerization, in agreement with our results (Zeidan et al., 2017).

With regard to EPS-producing *L. paracasei* strains, most of the studies report the monosaccharide composition of EPS synthetized in MRS or chemically defined medium added with different sugars. In general, EPS from *L. paracasei* strains are heteropolysaccharides mainly composed by glucose, mannose and galactose, along with some minor sugars such as fucose, xylose, rhamnose, arabinose, N-acetylgalactosamine, galacturonic and glucuronic acid, depending on the strain (Hee et al., 2011; Liu et al., 2013; Balzaretti et al., 2017; Bhat and Bajaj, 2019; Xiao et al., 2021; Zhang et al., 2021; Amini et al., 2022; Wang et al., 2022). In agreement with our results, EPS produced by *L. paracasei* DG (Balzaretti et al., 2017), *L. paracasei* KB28 (Hee et al., 2011) and *L. paracasei* KL1 (Liu et al., 2013) have also shown the presence of rhamnose in their composition. The presence of this monosaccharide as a constituent of EPS from *L. paracasei* strains seems to be a common feature. Smokvina et al. (2013) analyzed the genome of *L. paracasei* strains from different niches, evidencing that most of the studied strains presented two cluster that contain genes that encode rhamnosyltransferases and enzymes involved in the conversion of D-glucose-1-phosphate into dTDP-L-rhamnose. In fact, it has been proposed that the presence of rhamnose in EPS is relevant with regard to their biological effect since several rhamnose-rich EPS produced by species of *Lacticaseibacillus* have the ability to stimulate the production of proinflammatory cytokines by antigen-presenting cells (Balzaretti et al., 2017).

On the other hand, little is known about monosaccharide composition of EPS produced by *L. paracasei* strains in milk. Recently, Li et al. (2020) reported an HePS from *L. paracasei* H9 produced in milk that was composed by mannose, glucose, galactose and glucuronic acid. When analyzing the monomer composition of EPS produced by LAB in milk, the presence of glucose and galactose and their amino derivatives can be evidenced in most biopolymers, which is expected considering that lactose constitutes the main sugar present in milk (Zhu et al., 2019; Bachtarzi et al., 2020; You et al., 2020a, 2020b). These results are in line with HePS of *L. paracasei* CIDCA strains studied in the present work.

The characteristics of EPS produced by LAB are highly dependent on culture conditions, including temperature, medium composition and incubation time (Ibarburu et al., 2015). In a previous work, the EPS produced by these *L. paracasei* CIDCA strains in MRS agar was evaluated (Bengoa et al., 2018a). When comparing the molecular weight distribution of EPS produced in milk with those obtained in MRS, it can be clearly evidenced that they are composed by different fractions, showing the influence of the culture medium on the polymer characteristics. Interestingly, the temperature influence on the molecular weight distribution of EPS was equivalent both in MRS (Bengoa et al., 2018a) and milk, showing that low temperatures increase the proportion of high molecular weight fractions, favoring EPS polymerization. Furthermore, in the case of HePS produced by *L. paracasei* CIDCA 83123 and CIDCA 83124 in milk, it was evidenced that temperature also induced changes in the monosaccharide composition. In contrast to our results, Khanal and Lucey (2018) studied the effect of temperature fermentation in the molecular weight of EPS synthetized by two *Streptococcus thermophilus* strains and no significant changes in molecular weight were observed. Many studies have evaluated the effect of different culture conditions (incubation time and temperature, carbon, nitrogen, and mineral sources) on EPS yield (Amiri et al., 2019; Zhang et al., 2020; Chen et al., 2022). However, the influence of these factors on EPS characteristics, including molecular weight distribution and monosaccharide composition, has not been extensively studied. In fact, to our knowledge, the present work constitutes the first report of the effect of temperature fermentation on monosaccharide composition of EPS produced by LAB.

The structural diversity of EPS produced by *L. paracasei* provides a variety of rheological properties that can be exploited for diverse commercial applications in the food or medical industry. Moreover, the impact of culture conditions on EPS characteristics allows obtaining products with different technological properties simply by modifying the fermentation temperature. Acid milk gels obtained with *L. paracasei* strains at three fermentation temperatures presented a non-Newtonian flow behavior. Growth temperature did not significantly affect the apparent viscosity or the shear-thinning flow behavior of acids gels obtained with *L. paracasei* CIDCA 8339 and CIDCA 83123. This indicates that the changes on molecular weight distribution induced with temperature in the EPS synthesized by these strains did not generate a significant impact on the viscosity of the fermented product. On the other hand, acid gels obtained with *L. paracasei* CIDCA 83124 presented a significant increase in viscosity when temperature decreased, which is in line with the increment in the proportion of the high MW fraction. Moreover, differences in hysteresis area of the flow curves were also observed. These results are in agreement with those previously reported that indicated the relationship between EPS structure and its techno-functional properties. Bachtarzi et al. (2020) compared EPS produced by *L. plantarum* strains and demonstrated a correlation between a ropy phenotype and the presence of a high MW fraction, which also contributed to the viscosity of the fermented product.

Comparing flow curves and viscosity of the acid gels obtained with *L. paracasei* CIDCA strains at a given temperature, differences were observed. When grown at 30°C, the three strains produced the same amount of EPS indicating that, although the amount of EPS produced *in situ* is one of the factor affecting rheological properties, the physicochemical properties of the biopolymer are also of great relevance since the structure of EPS may significantly affect its interaction with milk proteins (Ruas-Madiedo et al., 2009). Considering the molecular weight distribution of the EPS produced by the three *L. paracasei* strains, the lower viscosity values of acid gels obtained with CIDCA 8339 correlated with the structural characteristic of the EPS as the high MW fraction produced by this strain is smaller than the corresponding high MW fraction produced by CIDCA 83123 and CIDCA 83124. On the other hand, *L. paracasei* CIDCA 83123 and CIDCA 83124 produce EPS that have similar molecular weight distribution, but their acid gels present different viscosity and flow behavior. While acid gels obtained with *L. paracasei* CIDCA 83123 have low apparent viscosity with flow curves that fit the Oswald de Waele model, acid gels obtained with CIDCA 83124 strain fit the Carreau-Yasuda model. Acidification rate along with fermentation temperature are factors that affect the texture of acid gels (Cobos et al., 1995; Lucey and Singh, 1997; Ruas-Madiedo and Zoon, 2003). Acidification rate determines the aggregation of casein micelles to form acid gels, together with the *in situ* production of EPS that may differentially affect protein network depending on the polymer physicochemical properties (Mende et al., 2016). Observing acidification curves for both strains at a specific temperature, it can be clearly evidenced that CIDCA 83124 strain has higher growth and acidification rates, which could be one of the factors that explain the differences evidenced in acid gels obtained with these two strains. Furthermore, these HePS could have different ramification degree that determines flow properties and macromolecules interaction.

Another important feature ascribed to bacterial EPS are their health-promoting properties since they are one of the molecules that may act as effectors of the biological role of probiotic microorganisms (Caggianiello et al., 2016; Laiño et al., 2016). One of the health effects attributed to EPS produced by probiotics is their prebiotic potential acting as compounds that can be selectively fermented by gut microbiota leading to the production of bioactive metabolites such as short chain fatty acids (acetate, propionate, and butyrate) (Ale et al., 2020; Sabater et al., 2020). This was the case of EPS produced by *L. paracasei* CIDCA 8339 and CIDCA 83124 in milk at 30°C, which have the ability to be metabolized by infant fecal microbiota differentially modifying the microbial populations as well as their metabolic activity (Bengoa et al., 2020a). While fermentation of EPS_8339_ increased propionate and butyrate levels, fermentation of EPS_83124_ only raised butyrate levels indicating that EPS molecular weight or monomer composition have a crucial role in the stimulation of gut bacteria growth and activity.

On the contrary, the results obtained in the present work with regard of *Salmonella enteritidis* invasion to intestinal epithelial cells in the presence of crude EPS, demonstrated that this effect was not dependent on the biopolymer structure since both EPS tested performed a similar barrier effect at the highest concentration. It has been previously evidenced that *L. paracasei* CIDCA 8339 and CIDCA 83124 grown in MRS medium can decrease *Salmonella* invasion. With regard to the mechanism involved, both strains inhibited *Salmonella* invasion by interaction with the pathogen meanwhile, only CIDCA 8339 protects through a barrier effect (Zavala et al., 2016). Therefore, it can be consider that EPS is not the only factor involved in epithelium protection from pathogen damage.

## 5. Conclusion

The present results demonstrate that temperature affects the growth and acidification rate of *L. paracasei* CIDCA strains affecting the time require to form the acid gel. At this point, an equivalent concentration of the three lactobacilli is obtained in the product without changes in organic acids levels. Given that *L. paracasei* CIDCA 83123 has low growth rates at all temperatures assayed, it could be considered the least attractive strain for its application at an industrial level. On the other hand, the ability of CIDCA 8339 and CIDCA 83124 strains to grow well in a wide range of temperatures could be relevant for the industry, since it enables their application in the development of a wide variety of foods without limitations in the product manufacturing conditions. Furthermore, milk fermentation with *L. paracasei* CIDCA 83124 at 20 or 30°C would constitute an alternative to improve the rheological properties of the product. The production *in situ* of EPS will also contribute to the healthy properties attributed to the fermented milk obtained with these strains by promoting the protection against *Salmonella* infection.

In light of these results, it can be concluded that the selection of the EPS-producing strain along with the appropriate fermentation conditions could be an interesting strategy to improve the technological properties of these *L. paracasei* fermented milks with potential health benefits. Deepening the knowledge of EPS structure of these strains would contribute to maximally exploit them for food application.

## Data availability statement

The original contributions presented in the study are included in the article/Supplementary material, further inquiries can be directed to the corresponding author.

## Author contributions

AB contributed in study design and conception, and performed experimental work, data interpretation, and manuscript writing. AP and MD participated in data discussion and manuscript revising. GG and AA participated in study design and conception, funding, and manuscript revising. All authors contributed to the article and approved the submitted version.

## Funding

The present work was supported by CONICET (PIP 2786), Universidad Nacional de La Plata (UNLP 18/X813), ANPCyT (PICT 2020–03973 and PICT 2020–3239), and the Basque Government (IT1662-22 and PIBA 2020_1_0032).

## Conflict of interest

The authors declare that the research was conducted in the absence of any commercial or financial relationships that could be construed as a potential conflict of interest.

## Publisher’s note

All claims expressed in this article are solely those of the authors and do not necessarily represent those of their affiliated organizations, or those of the publisher, the editors and the reviewers. Any product that may be evaluated in this article, or claim that may be made by its manufacturer, is not guaranteed or endorsed by the publisher.
