# Supplementary material for: Exopolysaccharide-producing Lacticaseibacillus paracasei strains isolated from kefir as starter for functional dairy products
Source: Front Microbiol. 2023 Feb 24;14:1110177. doi: 10.3389/fmicb.2023.1110177 (PMC9998950; doi:10.3389/fmicb.2023.1110177)
Supplement: Supplementary file 1 [file Data_Sheet_1.pdf]

# *Supplementary Material*

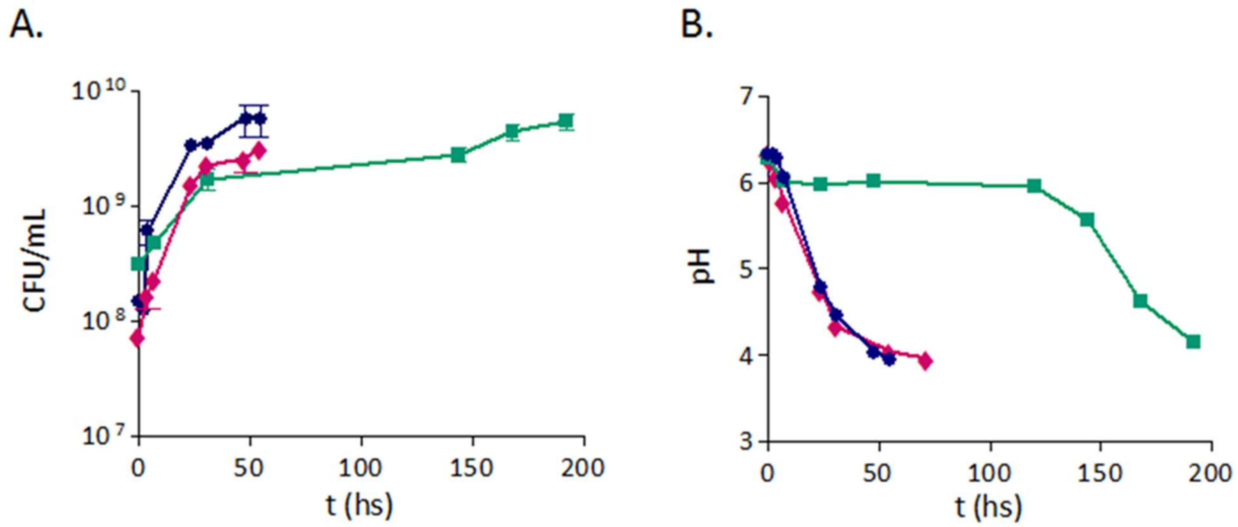


**Supplementary Figure 1.** Growth **(A)** and acidification **(B)** kinetics of *L. paracasei* CIDCA 8339 (♦), CIDCA 83123 (▪) and CIDCA 83124 (●) in milk at 20°C.

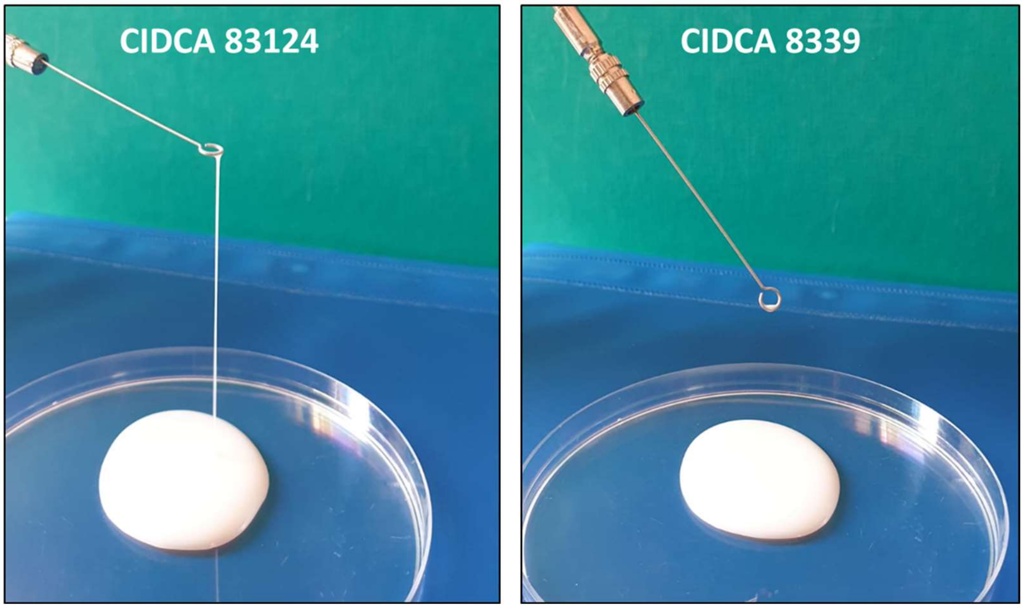


**Supplementary Figure 2.** Fermented milk with *L. paracasei* CIDCA 83124 (ropy character) and CIDCA 8339 (non-ropy character) at 30°C. Ropy character of fermented milk was defined by the formation of a filament between the fermented milk and a sterile loop.

**Supplementary Table 1.** Parameters obtained from Ostwald de Waele model corresponding to milks fermented with *L. paracasei* CIDCA 8339 and CIDCA 83123.

| *L. paracasei* | Temperature | | Up curve | | | | Down curve | | | |
|---|---|---|---|---|---|---|---|---|---|---|
| | | | k | n | $Chi^2$ | r | k | n | $Chi^2$ | r |
| CIDCA 8339 | 20 °C | A | 3.68 | 0.15 | 1.82 | 0.99 | 0.63 | 0.42 | 7.73 | 0.99 |
| | | B | 5.32 | 0.10 | 2.90 | 0.97 | 0.70 | 0.41 | 8.26 | 0.99 |
| | 30 °C | A | 6.92 | 0.08 | 5.14 | 0.93 | 0.71 | 0.43 | 10.41 | 0.99 |
| | | B | 8.27 | 0.05 | 4.84 | 0.86 | 0.71 | 0.43 | 10.42 | 0.99 |
| | 37 °C | A | 8.67 | 0.05 | 3.86 | 0.89 | 0.73 | 0.42 | 9.87 | 0.99 |
| | | B | 9.96 | 0.03 | 4.67 | 0.75 | 0.77 | 0.42 | 10.88 | 0.99 |
| CIDCA 83123 | 20 °C | A | 1.84 | 0.25 | 0.44 | 0.99 | 0.36 | 0.47 | 10.85 | 0.98 |
| | | B | 1.71 | 0.26 | 0.18 | 0.99 | 0.35 | 0.48 | 10.84 | 0.98 |
| | 30 °C | A | 6.10 | 0.10 | 5.50 | 0.95 | 0.48 | 0.48 | 11.13 | 0.99 |
| | | B | 6.15 | 0.10 | 5.91 | 0.94 | 0.47 | 0.48 | 10.65 | 0.99 |
| | 37 °C | A | 7.88 | 0.05 | 4.80 | 0.86 | 0.56 | 0.46 | 8.99 | 0.99 |
| | | B | 8.80 | 0.04 | 4.95 | 0.82 | 0.61 | 0.45 | 9.32 | 0.99 |

k: consistency index (Pa $s^n$); n: flow index

Flow index (n) of acid gels obtained by acidification with glucono δ lactona (GDL) = 0,8 ± 0,01 and consistence index k = 0,04±0,02

**Supplementary Table 2**. Parameters obtained from Carreau-Yasuda model corresponding to milks fermented with *L. paracasei* CIDCA 83124

| ***L. paracasei*** | **Temperature** | | **Carreau Yasuda model** | | | | | | |
|---|---|---|---|---|---|---|---|---|---|
| | | | **$\eta_0$** | **$\eta_\infty$** | **λ** | **n** | ***a*** | **Chi²** | **r** |
| CIDCA 83124 | 20 °C | A | 2.40 | 0 | 0.02513 | -0.18 | 2 | 406.0 | 0.99 |
| | | B | 2.49 | 0 | 0.02727 | -0.15 | 2 | 484.3 | 0.98 |
| | 30 °C | A | 1.86 | 0 | 0.02379 | -0.14 | 2 | 179.2 | 0.99 |
| | | B | 2.43 | 0 | 0.02659 | -0.13 | 2 | 215.7 | 0.99 |
| | 37 °C | A | 1.46 | 0 | 0.02789 | 0.03 | 2 | 18.8 | 0.99 |
| | | B | 1.35 | 0 | 0.02547 | 0.01 | 2 | 24.1 | 0.99 |

$\eta_0$: initial viscosity (Pa.s); $\eta_\infty$: viscosity to infinite time (Pa.s); n: flow behavior index; λ: time parameters ($s^{-1}$); *a*: constant (non-dimensional).

**Supplementary Table 3.** Apparent viscosity (measured at 300 $s^{-1}$) and thixotropy of milks fermented with *L. paracasei* CIDCA strains at different temperatures.

| *L. paracasei* | Temperature | | η at $300s^{-1}$ (mPa.s) | Thixotropy ($Pa.s^{-1}$) |
|---|---|---|---|---|
| CIDCA 8339 | 20 °C | A | 28.83 | $1.18 \times 10^3$ |
| | | B | 31.60 | $1.41 \times 10^3$ |
| | 30 °C | A | 35.42 | $1.71 \times 10^3$ |
| | | B | 36.14 | $1.86 \times 10^3$ |
| | 37 °C | A | 37.69 | $1.99 \times 10^3$ |
| | | B | 39.08 | $2.10 \times 10^3$ |
| CIDCA 83123 | 20 °C | A | 25.47 | $1.19 \times 10^3$ |
| | | B | 25.02 | $1.15 \times 10^3$ |
| | 30 °C | A | 35.17 | $2.01 \times 10^3$ |
| | | B | 34.58 | $1.98 \times 10^3$ |
| | 37 °C | A | 34.73 | $1.91 \times 10^3$ |
| | | B | 36.62 | $2.04 \times 10^3$ |
| CIDCA 83124 | 20 °C | A | 221.8 | $3.22 \times 10^4$ |
| | | B | 225.1 | $3.23 \times 10^4$ |
| | 30 °C | A | 198.80 | $2.85 \times 10^4$ |
| | | B | 234.3 | $3.36 \times 10^4$ |
| | 37 °C | A | 162.90 | $2.30 \times 10^4$ |
| | | B | 170.90 | $2.40 \times 10^4$ |

η: apparent viscosity

Apparent viscosity ($\eta_{app}$) at 300 $seg^{-1}$ of acid gels obtained by acidification with glucono δ lactona (GDL) =10 mPa.seg
